# Age-Related Changes in Post-Translational Modifications of Proteins from Whole Male and Female Skeletal Elements

**DOI:** 10.3390/molecules28134899

**Published:** 2023-06-21

**Authors:** Elizabeth Johnston, Michael Buckley

**Affiliations:** School of Natural Sciences, Manchester Institute of Biotechnology, University of Manchester, 131 Princess Street, Manchester M1 7DN, UK; elizabeth.johnston@manchester.ac.uk

**Keywords:** post-translational modifications, biological age estimation, forensic proteomics, rat bone

## Abstract

One of the key questions in forensic cases relates to some form of age inference, whether this is how old a crime scene is, when in time a particular crime was committed, or how old the victim was at the time of the crime. These age-related estimations are currently achieved through morphological methods with varying degrees of accuracy. As a result, biomolecular approaches are considered of great interest, with the relative abundances of several protein markers already recognized for their potential forensic significance; however, one of the greatest advantages of proteomic investigations over genomics ones is the wide range of post-translational modifications (PTMs) that make for a complex but highly dynamic resource of information. Here, we explore the abundance of several PTMs including the glycosylation, deamidation, and oxidation of several key proteins (collagen, fetuin A, biglycan, serum albumin, fibronectin and osteopontin) as being of potential value to the development of an age estimation tool worthy of further evaluation in forensic contexts. We find that glycosylations lowered into adulthood but deamidation and oxidation increased in the same age range.

## 1. Introduction

### 1.1. Proteomics in Forensics

Though DNA analysis is considered a gold-standard in forensics, it is highly prone to rapid degradation, either rendering specific DNA-typing impossible or reducing the possible information that can be garnered [1]. Proteins are much more stable than DNA [2], and their detection is considered greater into the archaeological and geological records [3]. Standard forensic practices are already being researched for their proteomic capabilities, such as with hair and bodily fluid analysis [1,4]. Moreover, proteomics is being employed to detect toxins and venom for the purpose of aiding criminal and food investigations [5,6,7]. The amount of forensically relevant information that can be obtained through proteomics is on par with that of genetics, even within single workflows [8,9]. Historically, this has focused on the identification of bodily fluids, as each has its own distinguishing proteome, with advancements allowing on-site identification of genetic material [10,11]; proteomic analysis even led to the discovery of the ABO blood groups [12]. Proteomics has since branched out into other tissues and forensically relevant areas, such as bone [13], which can be complementary to traditional methods of biological profiling by forensic anthropologists, such as post-mortem interval [13], sex [14] and age-at-death estimation [14,15] coming into recent prominence.

### 1.2. Bone Development

Bone begins development either through a cartilaginous model (endochondral ossification), producing cortical and diploic bone, or via mesenchymal tissue (intramembranous ossification), producing trabecular bone. Osteogenesis begins with a primary ossification centre, of which there can be multiple, followed by secondary centres. Primary ossification centres have mostly begun ossification before birth [16]. As development continues, between these centres, a cartilaginous layer allows bones to grow and eventually fuse, producing a complete, mature bone. This process is fairly predictable with little variability between individuals, and differences between males and females has been well recorded [16]. Modelling allows bones to take on their characteristic morphology through the functions of osteoblasts (bone-forming cells) and osteoclasts (bone-resorption cells) [17].

### 1.3. Bone Remodelling

Bone remodelling involves two processes: bone resorption, the removal of old bone, and ossification, the placement of new bone. This happens in small distinct areas at a time [18]. Remodelling happens in all bones, regardless of their initial development, whereby ~5% of cortical bone and ~25% of trabecular bone is remodelled every year in adults [16,19]. Remodelling can also occur as a response to physical traumas, such as mechanical loading and bone fracturing. It can be a slow process, taking four to five years per cycle [18]; with some skeletal elements experiencing much higher turnover rates than others. For example, ribs undergo turnover more so than any other skeletal element; this rate is often in response to metabolic and systemic factors as they are used by the body as a mineral reservoir, particularly calcium, rather than as a response to mechanical loading like most other bones [20]. Although this leads to ribs losing bone density the quickest in cases of osteoporosis, they do not present age-dependent mineral changes [18,20,21].

It is well known that bone remodelling slows down as humans’ age, whereby osteon canals have lower average diameters in older individuals, suggesting a lower proportion of active osteons compared to younger individuals, the latter experience more rapid remodelling [22]. Females and males may also have different processes that lead to bone loss in older age, with increased resorption and decreased formation, respectively [23].

Proteins are the foundation of all metabolic and regulatory processes within living beings [24], and as such are a key part to bone remodelling [25]. Osteoblasts, the bone-laying cells, are responsible for producing growth factor proteins, such as bone morphogenetic proteins (BMPs); that aid in osteoblast regulation and overall bone mass in adulthood [26]. BMPs control localized bone formation and even have clinical uses in stimulating bone formation in fracture patients [27]. This is just one example, but bone tissue contains thousands of proteins that make up the proteome [28].

Proteomic analyses are becoming ever more popular in the field forensics, especially as proteins have an extremely high longevity, particularly compared to that of DNA [2,14]. Although this high longevity can be attributed to the protein’s physical and chemical properties, proteins still undergo diagenesis, as the rapid pH decline after death within the body increases the rate of protein glycolysis and denaturation [29,30]. The move to more biological-based methods has arisen from the need for more accurate age estimation methods that are not as affected by observer error, nor as subjective [31]. As bone can endure a wide range of environments and external factors, it is often the most common material discovered in both forensic and ancient contexts [13].

Collagen is a popularly used protein biological marker in ‘ancient’ remains due partly to its longevity but also because it is the most abundant type of protein in the organic component of bone, making up 90% of organic material. This is largely type-I collagen [32,33], which is comprised of three polypeptide alpha chains; in mammals, there are two alpha-1 (I) chains and one genetically distinct alpha-2 (I) chain, which the latter shows the highest variance in amino acid sequence [34]. Collagen is part of the bone matrix used for mineral deposition that provides tensile strength, elasticity and rigidity to bone [23]; however, the mechanical properties of collagen, as well as bone overall, have been shown to change as humans’ age, with the addition of a much slower turn-over rate in elderly individuals contributing to their vulnerability to fracturing [23,35]. These changes include loss of overall bone mass, increased bone porosity, and loss of overall strength and bone elasticity, indicating an age-dependent change in the collagen network [35].

### 1.4. Post-Translational Modifications and Ageing

A number of post-translational modifications (PTMs) have been associated with age-related changes, namely deamidation and oxidation, as well as an accumulation in advance glycation end-products (AGEs) and cross-linking [36,37]. Oxidation, particularly of methionine, has also been shown to increase with age, as does the level of carbonylation, which is considered one of the best-studied age-related change [38,39]. Carbonylation has also been observed in higher amounts in older mice, compared to younger mice, and this process is responsible for higher protein aggregation [36]. In relation to biological age estimation, oxidation has been thoroughly studied, where it has been found to accumulate in proteins with age [40]. This is due to the build-up of reactive oxygen species (ROS) that are produced within cells in aerobic metabolism as animals age; or by indirect association with oxidative-stress by-products [41,42]. The high abundance of proteins within many biological systems means they are a ready target for oxidation [41]. Oxidative stress arises from when the amount of ROS exceeds the organism’s antioxidant system [42]. Despite this, minor amounts of oxidative stress have been discovered to be beneficial, by allowing cell adaptation to a variety of age-related pathologies, such as ischemia [43].

Deamidation of asparagine and glutamine has also been posited as a biological age marker due to their role in degenerative diseases, such as Alzheimer’s, and also as a marker for diagenesis and the post-mortem interval [44,45]. Glutamine deamidation happens at a slower rate to that of asparagine and so is more typically seen in low-turnover proteins; this makes glutamine more suitable for archaeological samples where asparagine deamidation was too rapid to be studied [45]. Glutamine deamidation analysis has also been suggested as a more sample-conservative method of dating bone, over traditional carbon dating [13,45]. Its rate is dependent on the pH of the environment, alkaline environments allow deamidation processes to flourish, however acidic environments will not show any deamidation in the bone at all for archaeological samples [45].

The collagen matrix within skeletal tissue is stabilized by covalent cross-linking between collagen molecules. These cross-links are formed via reactions between aldehyde molecules in lysyl oxidase protein monomers, forming β-1-galactosyl-hydroxylysine (Gal-Hyl) and α-1,2-glucosyl-galactosyl-hydroxylysine (Glc-Gla-Hyl) [31,46]. These cross-links provide the collagen network its plastic and elastic properties when under mechanical stress [47]. Hydroxylysine aldehyde cross-links are more commonly found within skeletal tissue, which are more stable than lysine aldehydes and have been found to accumulate with age in dentin, reportedly contributing to its longevity [48]. The opposite was seen in connective tissues, with the number of cross-links dropping as the tissue matured [48].

The production of AGEs has been studied regarding age estimation. Glycation is a non-enzymatic process that happens via the Maillard reaction; firstly, reducing sugars reversibly react with a free amino group resulting in a Schiff base. The Schiff base then undergoes an Amadori rearrangement, resulting in products that are stable. Amadori products accumulate in short- and long-lived proteins but also degrade into different carbonyl compounds that can then react with amino acids. They also undergo cleavage and covalent bonding to form stable adducts and cross-link-forming AGEs. After this, the process is irreversible and can take weeks to months to occur. AGEs accumulate and cause damage in long-lived proteins, such as collagen, which decreases its solubility and reduces collagen’s resistance to digestion by enzymes [47,49,50,51].

Whereas glycation is a non-enzymatic process of reacting reduced sugars with proteins, glycosylation is an enzyme-driven process of binding a carbohydrate, or glycan, to an organic compound, including proteins, creating a glycoprotein. The enzymes involved are usually nucleotide-specific glycosyltransferases [52]. Humans mainly have carbohydrates binding to either oxygen atoms (O-linked) or to nitrogen atoms (N-linked) on the peptide chain. O-glycosylation, or mucin-type glycosylation is the modification of serine and threonine residues in the Golgi apparatus, typically in bacteria, and there is no commonly agreed upon sequence for this type of glycosylation [53]. Peptide sequences can be attached to multiple O-linked glycans, causing up to 80% of their overall mass to be comprised of carbohydrates and are known as “mucins” [53]; however, N-linked glycosylation accounts for 90–95% of glycoproteins found in humans [53]. N-linked glycosylation happens in the lumen of the endoplasmic reticulum and Golgi apparatus, and plays a critical role in folding [53]. Protein turn-over has been demonstrated to be the biggest contributing factor to the accumulation of AGEs; this accumulation was shown to be much higher and quicker in cartilage collagen over skin collagen in humans, which corresponds to the faster turn-over rate of skin over cartilage [54]. The higher number of cross-links make the protein matrix of bone stiffer and increase its resistance to proteolysis, which subsequently affects remodelling; individuals with diabetes also display such changes [47]. Osteocalcin, the most abundant NCP in bone, is subjected to glycation via the formation of Schiff base and Amadori rearrangement also [49]. The abundance of glycated osteocalcin has been demonstrated to increase linearly and with age and when an individual reaches 60 years old; however, the study conducted did not define “adult years”, so this method may only be useful for excluding the elderly or juveniles and may not be precise enough to assess biological age outside of these parameters [49].

The aims of this study were to evaluate the potential of observing PTMs in LC-MS/MS-derived proteomic data of bone from across individuals of increasing age, in this case, using published data from a rat model spanning both males and females, for potential applications to ageing human forensic skeletal remains.

## 2. Results

### 2.1. Glycosylation of Lysine

The resulting proteins that displayed age-related changes in abundance of PTMs were as follows: collagen α1(I) (CO1A1), collagen α2(I) (CO1A2), collagen α1(II) (CO2A1), collagen α1(V) (CO5A1), collagen α1(XI) (COBA1), fetuin-A (FETUA), fibronectin (FINC), serum albumin (ALBU), biglycan (PGS1) and osteopontin (OSTP).

**Figure 1:**
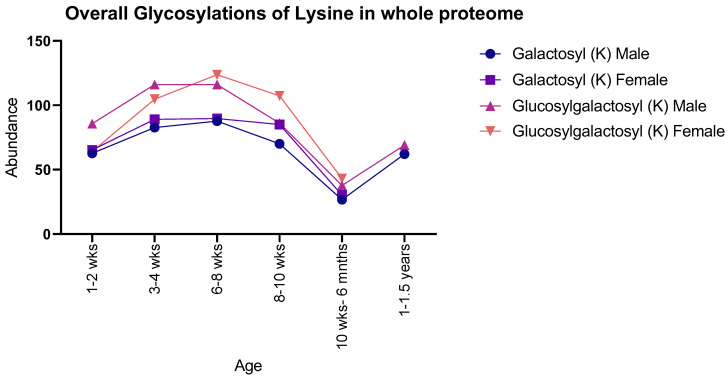
Number of detected glycosylations of lysine in whole rat proteomes for both male and female rats. Males and females showed similar numbers throughout life, with a peak abundance seen at 6–8 weeks.

Both males and females showed the same general pattern for galactosyl modifications with age when observing the proteome as a whole: there was an increase in both PTMs from 1–2 weeks to 8–10 weeks, with a sudden decrease at 10 weeks to 6 months, with 1–1.5-year-old males showing a subsequent increase. In contrast, glucosylgalactosyl modifications showed an increase between 1–2 weeks and 6–8 weeks, proceeded by a decrease until 10 weeks to 6 months, with 1–1.5-year-old males showing an increase (Figure 1). Moreover, instances of glucosylgalactosyl were generally higher in abundance than that of galactosyl for both sexes studied.

**Figure 2:**
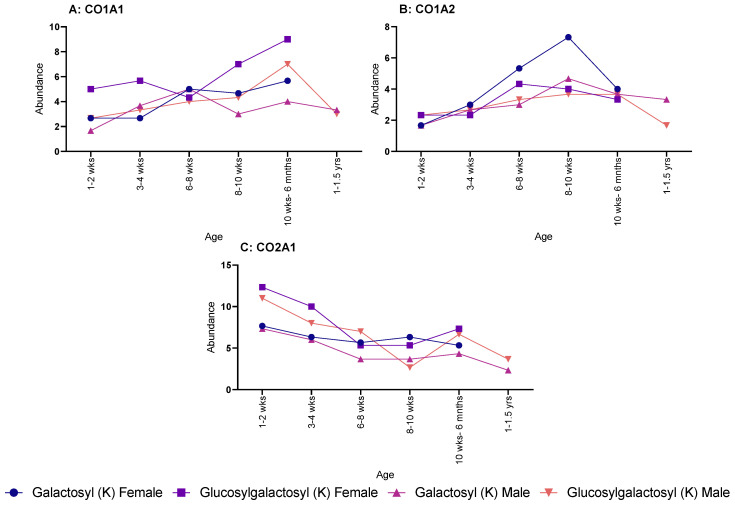
Age-related changes in galactosyl (K) and glucosylgalactosyl (K) modifications for both male and female rats. (**A**) Collagen-I-α-1 with males and females showing similar increase in abundance throughout life. (**B**) Collagen-I-α-2, showed a much shallower increase in abundance, except for galactosyl (K) in female, which rapidly peaked at 8–10 weeks. (**C**) Collagen-II-α-1, which steadily decrease in abundance for both male and females.

When looking at specific proteins, a different pattern was seen. CO1A1 showed a steadying increase in both galactosyl (K) and glucosylgalactosyl (K) for female rats, but no discernible pattern was seen over age for male rats (Figure 2A). The same sex difference was also seen in CO1A2 (Figure 2B), with males showing no pattern; however, female rats showed an increase throughout age for glucosylgalactosyl (K) but a decrease for galactosyl (K) modifications. Yet, CO2A1 showed a decrease in both PTMs for both males and females over age (Figure 2C).

### 2.2. N-Linked Glycosylations

**Figure 3:**
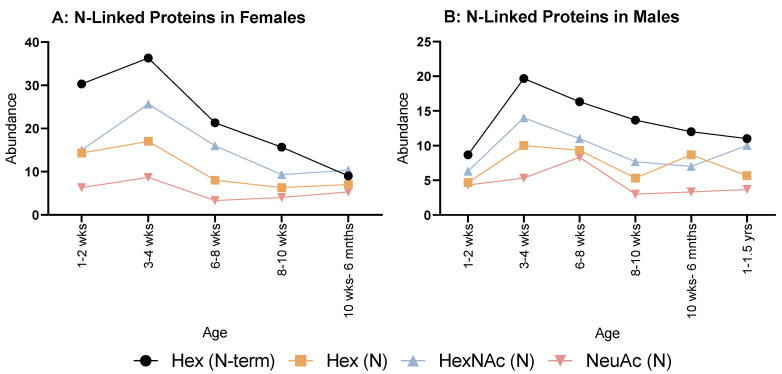
Abundance of N-linked glycosylations in the overall proteome in both female (**A**) and male (**B**) rats. (**A**) Female rats showed a relatively even but rapid decrease in all N-glycosylations from 3 to 4 weeks old. (**B**) Males also showed a similar decrease from 3 to 4 weeks, but less rapid than females.

When looking at N-linked glycosylations, females (Figure 3A) overall showed an increase from 1–2 weeks to 3–4 weeks, then decreased at 6–8 weeks for all. Hex (N) and NeuAc (N) showed a minimal, steady increase until 10 weeks to 6 months. Hex (N-term) and HexNAc (N) had minor decreases until 8–10 weeks, then HexNAc (N) plateaued, but Hex (N-term) decreased again at 10 weeks to 6 months.

As for males (Figure 3B), all modifications followed the same pattern of decreasing then increasing at 6–8 weeks, Hex (N) and NeuAc (N) reached their lowest number of instances at 8–10 weeks. Whereas for Hex (N-term) and HexNAc (N), this was reached at 10 weeks to 6 months with an increase at 6 months to 1 year old; however, NeuAc (N) displayed a steady increase after 8–10 weeks for all remaining ages.

### 2.3. O-Linked Glycosylations

**Figure 4:**
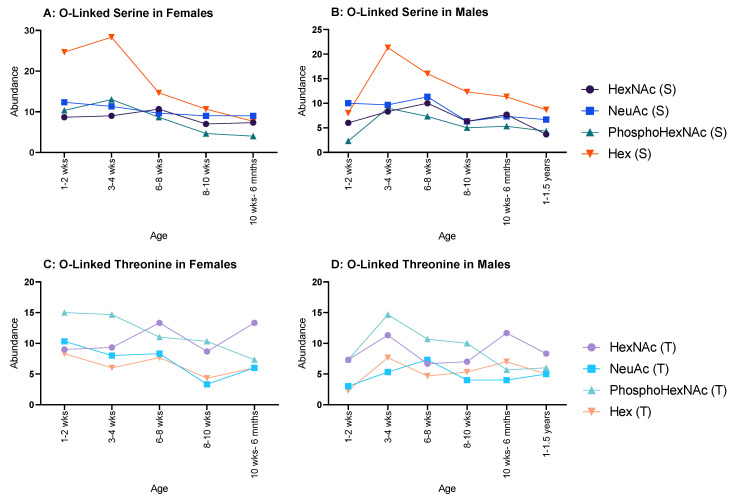
Abundance of O-linked glycosylations in the overall proteome for male and female rats. O-linked glycosylations are present on serine and threonine amino acids. (**A**,**B**) serine-related O-linked glycosylations in females and males, respectively, and both showed a decrease throughout life, but females began with an initially higher abundance in Hex(S) than males; and (**C**,**D**) threonine-related O-linked glycosylations in females and males, respectively, which showed a steady decline for both sexes.

In females, all serine glycosylations (Figure 4A) increased between 1–2 weeks and 3–4 weeks and decreased at 6–8 weeks, after which a minimal decrease was observed. Hex (T) and HexNAc (T) both increased between 1–2 weeks and 3–4 weeks, but NeuAc (T) and PhosphoHexNAc (T) only showed minor decreases between the same ages. HexNAc (T), NeuAc (T) and PhosphoHexNAc (T) all decreased until 8–10 weeks, then increased at 10 weeks to 6 months. Hex (T) increased until 8–10 weeks and decreased at 10 weeks to 6 months (Figure 4C).

Moreover, in males HexNAc (S) and NeuAc (S) followed a similar pattern, decreasing from 1–2 weeks to 3–4 weeks, increased at 6–8 weeks, decreased again at 8–10 weeks, and increased again at 10 weeks to 6 months (Figure 4B); however, HexNAc (S) decreased again at 1–1.5 years, whereas NeuAc (S) carried on increasing through the remaining ages. Hex (S) and PhosphoHexNAc (S) steadily increased from 1–2 weeks to 6–8 weeks and decreased to 8–10 weeks. Hex (S) decreased again at 10 weeks to 6 months, followed by a minimal increase at 1–1.5 years, while PhosphoHexNAc (S) showed a minimal, but steady increase from 8–10 weeks to 1.15 years. PhosphoHexNAc (T) showed a general increase throughout life. HexNAc (T) decreased from 1–2 weeks to 6–8 weeks, then rapidly increased until 10–6 months and then plateaued. NeuAc (T) oscillated between decrease–increase from 1–2 weeks to 8–10 weeks, then steadily increased thereafter. Hex (T) started relatively high, then decreased minimally until 8–10 weeks, then decreased rapidly at 10 weeks to 6 months and began to increase afterwards (Figure 4D).

When looking at individual proteins, not any one protein displayed an age-related change in either type of glycosylation.

### 2.4. Other Post-Translational Modifications

Some protein displayed more age-related changes in the number of PTMs than others, particularly collagen and its number of glycosylations; however, no one protein showed age-related changes across all PTMs selected, with collagen type I-α1 and α2 showing the most age-related changes. The changes in the number of PTMs were also not often consistent throughout life, with peaks and troughs in instances.

**Figure 5:**
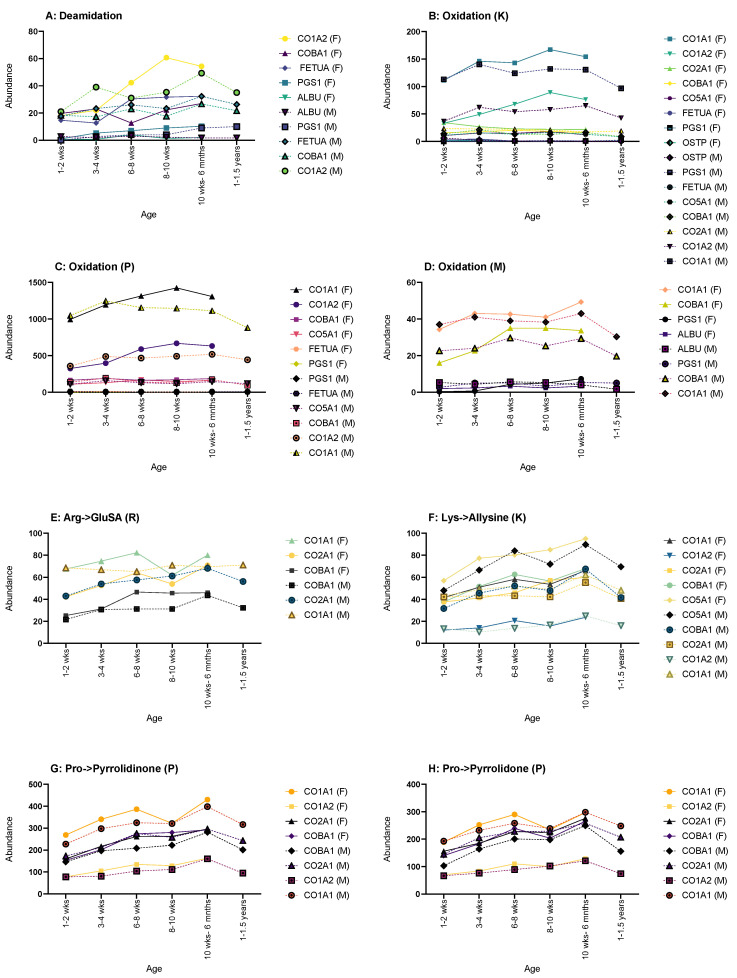
Graphs showing the proteins that displayed changes in abundance of PTMs across age. (**A**) Deamidation CO1A2, COBA1, FETUA, PGS1 and ALBU showed such a change. (**B**) Oxidation of lysine changes showed changes in CO1A1, CO1A2, CO2A1, COBA1, CO5A1, FETUA, PGS1 and OSTP, thus showing the highest number of proteins affected. (**C**) Oxidation of proline showed changes in CO1A1, CO1A2, COBA1, CO5A1, FETUA, PGS1, FETUA and PGS1. (**D**) Oxidation of methionine showed changes in CO1A1, COBA1, PGS1 AND ALBU. (**E**) Arg->GluSA had changes in CO1A1, CO2A1 AND COBA1. (**F**) Lys->Allysine had changes in CO1A1, CO1A2, CO2A1, COBA1 AND CO5A1. Pro->pyrrolidinone (**G**) and Pro->pyrrolidone (**H**) had the same proteins showing age-related changes: CO1A1, CO1A2, CO2A1 and COBA1. Graphs showing PTMs with proteins that showed no age-related change can be found in Supplementary Material Figure S1.

Deamidation often showed most of its age-related changes in both sexes, such as in fetuin-A, CO1A2, COBA1 and PGS1 (Figure 5A). Fetuin-A and COBA1 showed a steady increase for both males and females between 1–2 weeks to 10 weeks- 6 months, with males showing a decrease up to 1–1.5 years. CO1A2 also showed an increase for both sexes up until the oldest age sets in either sex, where a decrease was then shown. PGS1 also showed a steady increase throughout life for both males and females; however, only males showed any age-related changes in deamidations of serum albumin with a minimal decrease over time.

Oxidation of lysine (K) only showed age-related changes for both sexes in CO1A2 and CO2A1, the former showing an increase until the oldest age set followed by a decrease; the latter showed a decrease through life (Figure 5B). CO1A1, PGS1 and OSTP only displayed changes in males, with CO1A1 and PGS1 showing an increase in oxidation (K) and OSTP showing a decrease throughout. In females, COBA1 and CO5A1 had increases of oxidation (K) instances over time, and fetuin-A showed a decrease.

Oxidation of proline (P) showed no male-only age-related changes in any of the observed proteins (Figure 5C). Both sexes shared changes in CO1A1, CO1A2 and PGS1, with all three showing an increase throughout life, but CO1A1 and CO1A2 did show a decrease at the oldest age range for both sexes. Females show sex-only changes in fetuin-A, with a decrease over time, and with COBA1 and CO5A1 both showing an increase.

Only females showed any age-related changes in the oxidation of methionine (M) (Figure 5D). CO1A1, COBA1 and PGS1 all displayed an age-related increase in instances, as did ALBU until the oldest age range where a decrease was seen.

Arginine to glutamic semialdehyde (Arg->GluSA (R)) had very few changes (Figure 5E). Both sexes showed an increase in instances throughout life in CO2A1 and COBA1; however, males did show a decrease for both at 1–1.5 years. Males also showed one addition change in Arg->GluSA (R) in CO1A1, with a steady increase up until 8–10 weeks, followed by a general plateau after.

Lysine to allysine (Lys-Allysine (K)) had the most age-related changes in female rats (Figure 5F). CO1A1, CO1A2, CO2A1 and COBA1 all showed a general increase over time. Both sexes showed an increase in CO5A1, but once again, males showed a decrease at 1–1.5 years.

Both sexes showed age-related changes in pro-pyrrolidinone (P) for CO1A1, CO2A1 and COBA1 with an increase over age, with males showing a decrease for all at 1–1.5 years (Figure 5G). Males also showed changes in CO1A2 with an increase until 1–1.5 years where a decrease is then seen.

Pro-pyrrolidone (P) showed the most changes in males (Figure 5H). CO1A2, CO2A1 and COBA1 showed typical change in males, with an increase followed by a decrease at 1–1.5 years. Both sexes showed an increase in CO1A1, with males decreasing at 1–1.5 years.

Interestingly though, fibronectin appeared to apply to the previous criteria, and when examining individual proteins, no age-related changes in PTMs were observed.

## 3. Discussion

Glycosylations are notoriously difficult to analyse, and not any one method has been universally shown to be optimized for glycan analysis [55,56]. Between the two modifications applied in this study, glycosyl-galactosyl-hydroxylysine is noted to be in higher abundance within bone, whereas galactosyl-hydroxylysine is more common in soft tissues [46], which is reflected in this study with no apparent sex-related differences in number of PTMs. Supporting previous research [57], collagen cross-linking increases with age, and this was reflected in our study with female rats for collagen-I, but not observed in male rats, whereas conversely, both sexes showed a decrease in them for collagen-II. The human skeleton is constantly and gradually renewed throughout life through remodelling, the balanced activity between osteoclasts (the bone-resorbing cells) and osteoblasts, where modelling defines bone shape and development, and mainly occurs in adulthood as a response to prolonged physical strains [58]. In rats it was long thought that modelling was the primary bone activity throughout, as secondary ossification centres are not seen, and remodelling is limited in cortical bone, with trabecular bone showing the majority of remodelling [59]; however, studies have shown that rats steadily move over to remodelling well into adulthood. The age this happens can be different between the proximal and distal bone portions and between sexes. For example, in the tibia, this transition happens in the proximal end as late as 15 months and in the distal end as early as 3 months of age [60]. This may account for the differences shown between the younger and older ages, as age’s pre-8–10 weeks have yet to reach sexual maturity and the skeleton has not completed the modelling phase; ages 10 weeks-1.5 years may better reflect the transition from modelling to remodelling.

Ratios between different types of N-glycosylations have been established to change with age in humans as well as the presence of particular glycans or the position of them, particularly after middle age when biological functions begin to change [61,62]. Moreover, increases in N-glycosylations have also been observed in healthy individuals, and have been suggested to be an indicator of good health in old age [63]. This is linked to how glycosylations affect the overall structure and sometimes changes or inhibits the function of proteins [62]. Furthermore, some specific proteins have been studied for N-linked glycosylations, with disputes on whether an n-linked glycosylation even exists, such as on osteopontin [64].

This study only included whether a glycosylation is present, whereby the method of extraction and analysis could not provide information on the structure of the glycan, which may provide insight into why looking at individual proteins does not reflect biological age. Additionally, some studies suggest that the increased proportion of non-glycosylated proteins over glycosylated ones is a sign of increased age, such as with biglycan [65]. If individual proteins are of interest, specific analysis and enrichment is needed to observe any age-related changes.

O-linked glycosylations are difficult to analyse, due to their high heterogeneity and lack of need for a specific amino acid sequence for binding sites [66]. This study may only reflect a small percentage of present O-glycosylations as the method undertaken does not focus on separation of glycans. Most methods that utilize ESI-MS first apply HPLC or anion exchange MS before using ESI [66,67]. The overall lack of observable glycosylations in these samples may not be indicative of a lack of them at all. The before-mentioned limitations in sample preparation and analysis have highlighted the difficulties in analysing glycosylations, without rapid and reliable methods of extraction and analysis, particularly in bone.

Mono-oxidation of methionine is suggested to increase with age due to its susceptibility to ROS but is also reversible by methionine sulfoxide reductase [68]. Looking for methionine sulfoxide residue over its reduction back to methionine may be a more reliable age indicator than looking for just methionine oxidation alone [69]. Methionine oxidation-reduction is cyclical, the reduction that was seen in CO1A1 and osteopontin might be reflective of the reduction phase, and levels may increase once again in elderly years due to the ability to reduce methionine sulfoxide to methionine, but more research including elderly samples is needed to observe this. Post-mortem oxidation of proteins is known to occur as processes within the body turn from aerobic to anaerobic [70], this was limited by keeping samples frozen between processing, but this may present an issue in forensic cases were remains can be left exposed to the elements, or not discovered for several years and must be kept in mind if looking at PTMs.

Carbonylation on the other hand is irreversible and can result in unfolding of the protein structure [71]. Jana et al. [72] suggested that rats only showed age-dependent changes in carbonylation in albumin when only looking at plasma proteins, and this was observable in mice and rhesus monkeys when using SDS-PAGE. Tanase et al. [36] also concluded that carbonylation of arginine to glutamic semialdehyde and proline to pyrrolidinone or pyrrolidone causes protein aggregation and saw an increase in carbonylation PTMs; however, their studies focused on bone marrow of mice, and they induced oxidative stress in younger samples to observe aggregates.

Research into oxidative changes in bone is limited but is linked to several advanced-age related diseases, such as osteoporosis. This is caused by ROS-induced cell-death of osteoblasts and osteoclasts, the cells responsible for old bone resorption and new bone lay down, disrupting the ratio between the two leading to overall loss in bone density [73]. In order to study oxidative-stress-related diseases in rats, this has to be artificially induced, as rats can be resistant to such modifications [74]. As this study observes proteins from the tibia, this may account for the lack of oxidative PTM changes seen, as many of the rat ages studied are during the modelling phase in rats.

As previously stated, deamidation can occur within the laboratory during extraction and processing. In this study, to prevent lab-based deamidation as much as possible, EDTA was used at the demineralization stage, as this has been shown to limit these deamidations [75]. Deamidation has also been shown to open up the protein structure, making it more susceptible to proteolytic degradation, especially in high-turnover proteins [76]. In general, the rate of protein turnover within bone increases with age [77], leading to a higher instance of deamidation within many proteins. The results in this study confirm the increase in deamidation as age increases, at a steadier rate in female rats than male rats. Differences in deamidation sites between male and female mice has been noted before [78], showing that increased deamidation in mice was linked to a decrease in the toughness of cortical bone.

## 4. Limitations of the Study

How well a PTM profile from *Rattus norvegicus* aligns with a human PTM profile can only be validated through human data. Rats were used in this study as a human analogue due to difficulties in obtaining human samples that cover a similar age range, with replicates, and the same number of samples for both sexes.

Moreover, these samples were not enriched or prepared with any one PTM in mind for later analysis, though the charges allowed through during LC-MS/MS was to account for glycosylations. The results may differ if the extraction method is more tailored to specific proteins; however, samples may have to undergo multiple processes to obtain a wider profile, and this may not be available in forensic contexts, or a smaller profile may have to be produced with the most reliable PTMs chosen to be focused on.

## 5. Materials and Methods

### 5.1. Sample Preparation

Proteome data were taken from Johnston and Buckley [79]. In brief, whole rats (*Rattus norvegicus*) at 1–2 weeks, 3–4 weeks, 6–8 weeks, 8–10 weeks, and 6–12 months old for both sexes, and 1–1.5 years for males were macerated in 37 °C water for 4 days in individual beakers to allow maximum removal of soft tissues. Fused tibia and fibula were selected, with three triplicates at each age for both sexes having produced a sample set of 33 samples (5 age categories for females, 6 for males). Whole bones were selected to overcome ontological effects on results. The tibia and fibula were degreased in a solution of 83% chloroform and 17% methanol for 30 min, this solution was then disposed of, and a fresh solution of the same components and volume was added to the bones. This was then left overnight to fully degrease.

### 5.2. Protein Extraction

The dry bones were weighed, and a corresponding amount of 0.5 M tetrasodium ethylenediaminetetraacetic acid (EDTA) (pH 8.0, Life Sciences, Bedford, UK) was added to each bone at 4 °C for 18 h. The bones were removed from the EDTA, and the same amount of guanidine hydrochloric acid (GuHCl) was added and left at 4 °C for 18 h, producing the insoluble fraction. The insoluble fraction was focused on in this study as all proteins were being considered, and the EDTA fraction may contain NCPs, the amount of collagen in this fraction may mask other protein signals.

Ultrafiltration is carried out with 10 kDa molecular weight cut-offs centrifuge filters (MWCO; Sartorius, Goettingen, Germany) and filtered at 12,400 rpm. Next, the same volume of 50 mM ammonium acetate (AMAC) was added to the filters and filtered again at 12,400 rpm, this was repeated twice more. The proteins were then resuspended and collected off the filter with AMAC for reduction with 1 mM dithiothreitol (DTT) for 10 min at 60 °C. Then, alkylation was undertaken using iodoacetamide (IAM) and left in darkness for 45 min at room temperature. DTT was then added to prevent overalkylation. Trypsin was then added and left to digest for 18 to 22 h at 37 °C, then 1% TFA was added to cease digestion. The resulting digested sample was purified using Agilent OMIX C18 zip-tips, and the proteins were eluted with 50% acetonitrile (ACN) + 0.1% Trifluoracetic acid (TFA) and left to dry for 48 h. The dried samples were resuspended in 5% ACN/0.1% formic acid (FA) and analysed via LC-MS/MS.

### 5.3. Mass Spectrometry

LC-MS/MS were carried out using an UltiMate^®^ 3000 Rapid Separation LC (RSLC, Dionex Corporation, Sunnyvale, CA, USA) coupled to an Orbitrap Elite (Thermo Fisher Scientific, Waltham, MA, USA) mass spectrometer. Peptide mixtures were separated using a gradient from 92% A (0.1% FA in water) and 8% B (0.1% FA in acetonitrile) to 33% B in 44 min at 300 nL min^−1^ using a 250 mm × 75 μm i.d. 1.7 μM M-Class CSH C18 analytical column (Waters). Peptides were selected for fragmentation automatically by data-dependent analysis, with the top 6 ions selected per cycle. Fragmentation was achieved via CID. The *m/z* range for precursors was 350–1500 with a normalized collision energy of 35. The minimum signal threshold was 500 counts and an isolation width of 1 Da. All charge states were allowed except singly charged and unassigned charge states. Precursor ions were selected for fragmentation twice, with an exclusion window of 30 s. Precursor ions were measured in the Orbitrap with a resolution of 120,000 in profile.

### 5.4. Data Analysis

Each sample was searched using Mascot against SwissProt’s *Rattus* sequences with a decoy database. For each search, the fixed modifications included carbamidomethyl (C). *Rattus norvegicus* was cross-referenced with GlyConnect to determine the selected glycosylation PTMs for both O-linked and N-linked searches (Table 1). Due to the number of variable modifications required, these were run in multiple sets. Overall, these included galactosyl and glucosylgalactosyl (K), deamidation (NQ), oxidation (K/P/M), arginine-glutamic semialdehyde (R), allysine (K), pyrrolidinone (P) and pyrrolidone (P). The peptide charge option was set to 2+, 3+ and 4+, peptide tolerance to 5 ppm and MS/MS tolerance to 0.5 Da; this was to ensure glycosylations could be detected [80]. The Mascot searches for each sample were exported, providing a list of identified peptides for each protein and the position of the PTMs on each peptide.

The results were then manually searched for the variable modifications. The proteins were filtered by a mascot score of ≥11 as it was half the average score distribution of all the samples as PTMs were largely lost at higher scores. Each instance of each PTM per peptide was counted using a Visual Basic for Applications calculation that was manually coded in Microsoft Excel.

For a more focused view of PTMs, certain proteins were selected for individual analysis. The criteria for these chosen proteins were as follows: the number of instances for glycosylations was ordered into descending order for each medium adult sample (this was the median age for both sexes); the ordered list of peptides was checked for reoccurring proteins; and if three or more peptides for a protein was identified to contain at least one glycosylation, it was accepted for analysis. The instances for each PTM were counted for these selected proteins for every sample then averaged across each replicate. The PTM averages were normalized by the number of peptide matches for each of these proteins. 

## 6. Conclusions

In this study, rat bone proteomes of several distinct age categories were investigated to identify PTMs that could relate to age, and therefore have potential in forensic age-estimation cases. Our data found that this approach to map PTMs was limited partly by the modelling phase of the specimens analysed, i.e., all still within their initial bone development/growth stages; therefore, future work should focus more on the older phases of the lifecycle. However, this would be better conducted on other more appropriate human analogues reflecting the transition of modelling to remodelling phases, such as pigs or sheep. We also found the PTM signatures to be relatively sex dependent, but it was not distinct enough to be used as an approach in determining sex. Lastly, we observed that no single PTM appeared ideal on its own as a marker of age, and therefore, a suite of PTMs would be more appropriate to move towards establishing biological age.

## Figures and Tables

**Table 1:** List of post-translational modifications chosen for Mascot searches.

Glycosylation of Lysine	N-Linked Glycosylations	O-Linked Glycosylations	Other PTMs
Galactosyl (K)	Hex (N-term)	HexNAc (S)/(T)	Deamidation (NQ)
Glucosyl-galactosyl (K)	Hex (N)	NeuAc (S)/(T)	Oxidation (K)/(M)/(P)
	HexNAc (N)	PhosphoHexNAc (S)/(T)	Arg→GluSA (R)
	NeuAc (N)	Hex (S)/(T)	Lys→Allysine (K)
			Pro→Pyrrolidinone (P)
			Pro→Pyrrolidone (P)

## Data Availability

The mass spectrometry proteomics data have been deposited to the ProteomeXchange Consortium via the PRIDE repository with the dataset identifier PXD022055.

## Supplementary Materials

The following supporting information can be downloaded at: https://www.mdpi.com/article/10.3390/molecules28134899/s1, Supplementary Figure S1—Graphs of post-translational modifications with proteins that showed no age-related change.
