# Supplementary material for: Age-Related Changes in Post-Translational Modifications of Proteins from Whole Male and Female Skeletal Elements
Source: Molecules. 2023 Jun 21;28(13):4899. doi: 10.3390/molecules28134899 (PMC10343923; doi:10.3390/molecules28134899)
Supplement: Supplementary file 1 [file molecules-28-04899-s001.zip › molecules-2398103-Supplementary .pdf]

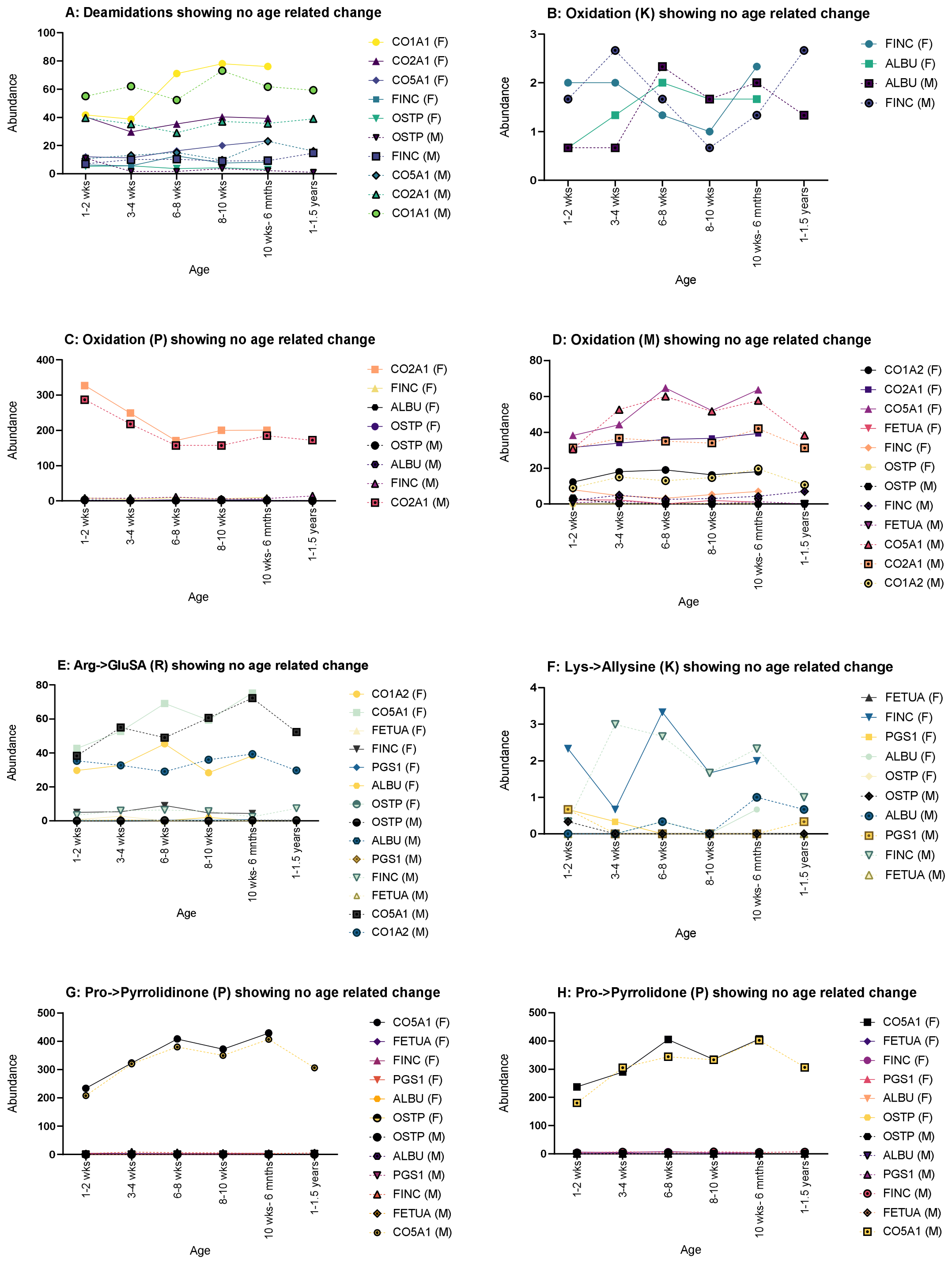

Supplementary Figure S1: (A) Proteins that showed no age-related changes in the number of deamidations. (B) Proteins that showed no age-related changes in oxidation (K). (C) Proteins that showed no age-related change in oxidation (P). (D) Proteins that showed no age-related change in oxidation (M). (E) Proteins that showed no age-related change in Arg-> GluSA (R). (F) Proteins that showed no age-related change in Lys-> Allysine (K). (G) Proteins that showed no age-related change in Pro-> Pyrrolidinone (P). (H) Proteins that showed no age-related change in Pro->Pyrrolidone (P).
